# Microstructural White Matter Changes in the Corpus Callosum of Young People with Bipolar Disorder: A Diffusion Tensor Imaging Study

**DOI:** 10.1371/journal.pone.0059108

**Published:** 2013-03-19

**Authors:** Jim Lagopoulos, Daniel F. Hermens, Sean N. Hatton, Juliette Tobias-Webb, Kristi Griffiths, Sharon L. Naismith, Elizabeth M. Scott, Ian B. Hickie

**Affiliations:** Clinical Research Unit, Brain and Mind Research Institute, University of Sydney, Camperdown, Australia; Baylor College of Medicine, United States of America

## Abstract

To date, most studies of white matter changes in Bipolar Disorder (BD) have been conducted in older subjects and with well-established disorders. Studies of young people who are closer to their illness onset may help to identify core neurobiological characteristics and separate these from consequences of repeated illness episodes or prolonged treatment. Diffusion tensor imaging (DTI) was used to examine white matter microstructural changes in 58 young patients with BD (mean age 23 years; range 16–30 years) and 40 controls. Whole brain voxelwise measures of fractional anisotropy (FA), parallel diffusivity (λ//) and radial diffusivity (λ⊥) were calculated for all subjects. White matter microstructure differences (decreased FA corrected p<.05) were found between the patients with BD and controls in the genu, body and splenium of the corpus callosum as well as the superior and anterior corona radiata. In addition, significantly increased radial diffusivity (p<.01) was found in the BD group. Neuroimaging studies of young patients with BD may help to clarify neurodevelopmental aspects of the illness and for identifying biomarkers of disease onset and progression. Our findings provide evidence of microstructural white matter changes early in the course of illness within the corpus callosum and the nature of these changes suggest they are associated with abnormalities in the myelination of axons.

## Introduction

Adult patients with Bipolar Disorder (BD) commonly recall that their symptoms commenced early in adolescence [1], [2]. Recent epidemiologic evidence confirms the emergence not only of depressive illness but also of adult-style manic and hypomanic episodes in the early and late adolescent periods [3]. This developmental period also coincides with the maximal myelination of several major white matter tracts including the corpus callosum (CC) [4]. To date most investigations of microstructural white matter changes in patients with BD have been conducted in patients who are in mid-life or who have persistent or recurrent disorders [1], [5]–[8]. These studies have identified a wide range of abnormalities including structural changes within the CC, cingulate as well as the inferior and superior longitudinal fasciculi [1],[5],[7],[8]. Moreover, a recent meta-analysis of diffusion tension studies [9] suggests two clusters of abnormality (on the right parahippocampal gyrus and subgenual cingulate cortex). Interestingly, only two studies have investigated adolescents with BD (albeit in relatively small cohorts). A study by Adler et al., found white matter changes within the superior-frontal white matter tracts [10] and recently Barnea-Goraly et al., reported more extensive white matter changes, as reflected by decreased FA, encompassing the corpus callosum, fornix, mid posterior cingulate gyrus as well as posterior regions encompassing the occipital corona radiata [11]. In addition to the aforementioned studies, several groups have also investigated white matter changes in young cohorts of healthy BD offspring who are considered to be at a greater risk of developing BD [11]–[14]. These young “at-risk” studies have provided new insights into the developmental trajectory of white matter maturation and how this process may go awry in people who go onto develop BD. A recent study by Versace et al., is particularly noteworthy as it reports age related white matter changes in otherwise healthy offspring of patients with BD and suggests diverging developmental trajectories across young people “at-risk” and controls [14]. This finding is corroborated by Sprooten et al. who similarly report significant white matter changes, in a larger “at-risk” cohort of young people [13].

Young persons with BD who are typically closer to their illness onset provide a unique window of opportunity for investigating this critical period (during adolescents or early adulthood) to identify core neurobiological characteristics of the illness (i.e. developmental markers or early illness biomarkers). Observed effects in these subjects at the emergence of the disorder are more likely to be independent of the deleterious effects of repeated illness episodes and other confounding factors such as prolonged exposure to treatments or secondary comorbidities. Neuroimaging studies of larger numbers of young patients with BD, early in the illness course, therefore, may assist identification of neurodevelopmental aspects of the disorder or markers of illness onset and progression.

Diffusion tensor imaging (DTI) is an established application of MRI that is sensitive to the microstructural organisation of white matter tracts. When reported as fractional anisotropy (FA), it provides insights into the role of structural (dis)connectivity at a much earlier stage of an illness, if not prior to its onset [15], [16]. Abnormalities to specific white matter tracts are increasingly being investigated as a means of identifying specific pathology, particularly within the early phases of disease process [11], [17]. FA is widely regarded a robust measure of white matter “organization” and indeed all of the aforementioned studies have reported abnormalities in FA. However, despite a confirmation of significant disruption to white matter integrity, little progress has been made delineating the underlying pathophysiology based solely from FA. Disruptions in white matter organization, (as reflected in reductions in FA) can result from various mechanisms including demyelination as well as discrete loss of axons. As such additional, complementary DTI metrics that distinguish between these two processes are essential. In this regard quantitative measures of parallel (λ//) and radial (λ⊥) diffusivity are also be obtained from DTI, and these measures describe water diffusion along (λ//) or across (λ⊥) axons thus providing information thought to reflect the integrity of axons or myelin, respectively. Parallel diffusivity is reportedly a measure of axon numbers and loss of axons are reflected in a decrease in this measure [18], [19]. Conversely, radial diffusivity characterizes the diffusion across the axon and myelin and thus disruptions in the myelin sheath are characterised by increased radial diffusivity [20]–[22].

The principal aim of this study was to determine whether there are characteristic microstructural white matter changes evident in young people in the earliest phases of BD. In accordance with the extant literature we hypothesised that young people with BD would exhibit focal changes within the corpus callosum as well as within white matter tracts connecting frontal and temporal regions including that previously have been reported as having reduced grey matter volume.

## Materials and Methods

### Participants

Fifty-eight young persons with BD (mean age = 23.03 years; range = 16 to 30 years) were recruited from an ambulatory care service for assessment and early intervention of mental health problems in young people [23], [24]. Those who participated are part of a broader longitudinal study of youth mental health at the Brain and Mind Research Institute (BMRI), Sydney, Australia. Control subjects (mean age = 24.05 years; range = 16 to 30 years) were recruited via advertisements in community newspapers as well as snowball techniques where controls were asked to suggest the name of two people (between the ages 16 and 30 years) who they thought may be interested in participating in the study. All control subjects were screened for psychiatric illness.

Subjects were also excluded if they were medically unstable (as determined by the treating psychiatrist), had a history of neurological disease (e.g. tumour, head trauma, epilepsy), medical illness known to impact cognitive and brain function (e.g. cancer, ECT in last 3 months), intellectual and/or developmental disability (a predicted IQ score <70) which prevented them from participating in the neuropsychological aspects of the concurrent studies [25] and insufficient English for testing or psychiatric assessment. Pre-morbid intelligence (‘predicted IQ’) was estimated on the basis of performance on the Wechsler Test of Adult Reading [26]. The Human Research Ethics Committee of the University of Sydney, Australia approved this study, and all participants gave prospective written informed consent for their clinical data to be used for research purposes. Written informed consent was obtained from the next of kin for all participants under 18 years of age.

An independent psychiatrist or trained research psychologist/neuropsychologist conducted a structured clinical interview to assign a DSM-IV [27] diagnosis, as well as to characterise the broader nature, history and clinical course of any mental health problems. The interview included the Hamilton Depression Rating Scale (HDRS, 17-item) [28] to quantify current (over the last 7 days) mood symptoms; the Brief Psychiatric Rating Scale (BPRS) [29] to quantify general psychiatric symptoms at the time of assessment, Young Mania Rating Scale (YMRS) [30]; and the social and occupational functioning assessment scale (SOFAS) [31]; where a patient’s functioning is rated from 0 to 100, with lower scores suggesting more severe impairment. Patients also completed the Kessler-10 (K-10) [32], a brief self-report instrument designed to detect psychological distress and affective caseness [33].

### Determination of Bipolar Syndrome Status

All subjects were assessed by a senior psychiatrist and, on at least one separate occasion by a neuropsychologist using our BMRI Structured Interview for Neurobiological Studies [34]. The interviews include rating the likelihood that the young persons had ever had a manic or hypomanic episode, or had an illness course consistent with a bipolar spectrum disorder. After completion of this detailed diagnostic process, subjects were assigned by consensus of the senior investigators (IBH and ES) to the “bipolar-type” category on the basis of: (i) a clinical diagnosis of at least one discrete episode of mania or hypomania (DSM-IV criteria); or (ii) an illness pattern consisting of periods of both elevated and depressed mood consistent with a bipolar spectrum disorder [35]. For those classed as having BD, 18 were classified as bipolar I (age of psychiatric onset: 16.8±3.2; illness duration: 5.6±3.9), 27 as bipolar II (age of psychiatric onset: 15.8±3.1; illness duration: 7.8±5.4) and 13 were bipolar spectrum disorder (age of psychiatric onset: 12.6±3.9; illness duration: 9.7±5.2). Thirty-nine percent of bipolar I patients had psychotic features compared to 19% and 8% of patients diagnosed as bipolar II and bipolar spectrum disorders, respectively (Table 1). All patients were receiving clinician-based case management and relevant psychosocial interventions at the time of assessment. Additionally, patients who were treated with psychotropic medications were assessed under ‘treatment as usual’ conditions, whereby their normal medications were not altered. At the time of assessment, 13% of patients were not taking any psychotropic medications; 43% were taking a second-generation anti-depressant, 63% an atypical antipsychotic medication and 39% were taking a mood stabiliser.

**Table 1:** Age of onset, duration of illness and the percentage of patients with psychosis for bipolar I, II and bipolar spectrum patients.

	Bipolar I (N = 18)	Bipolar II (N = 27)	Bipolar spectrum (N = 13)
Age of psychiatric onset	16.8±3.2	15.8±3.1	12.6±3.9
Duration of illness	5.6±3.9	7.8±5.4	9.7±5.2
Patients with psychosis (%)	38.90%	18.50%	7.70%

Statistical analyses were performed using SPSS for Windows 20.0. Group differences in demographic, clinical and neuropsychological variables were assessed with one-way analysis of variance (ANOVA) or chi-square tests where relevant. If equality of variance was compromised (according to Levene’s test) the corrected degrees of freedom and p-values were reported.

### MRI Acquisition

All imaging was performed on a 3T GE Discovery MR750 scanner (GE Medical Systems, Milwaukee, WI) at the BMRI imaging facility. Whole brain diffusion-weighted images where acquired using an echo planar imaging sequence with the following image parameters: repetition time (TR) = 7000 ms; echo time (TE) = 68 ms; Slice thickness = 2.0 mm; field of view (FOV) = 230×230 mm; acquisition matrix = 256×256; 69 gradient directions. Two images without gradient loading (b0 s/mm2) were acquired prior to the acquisition of 75 images (each containing 55 slices) with uniform gradient loading (b0 = 1000 s/mm2). In addition to diffusion-weighted images we also acquired T1-weighted structural images for the purpose of anatomical localisation.

### Tensor Calculations

All data was initially analysed using the FMRIB Software Library [FSL vers. 5.0; http://www.fmrib.ox.ac.uk/fsl] [36]. Firstly, the FDT toolbox was used to correct all data for spurious eddy current distortions as well as motion artifacts by applying affine alignment of each diffusion-weighted image to the first volume of the diffusion data without gradient (i.e., the b = 0 image). The Brain Extraction Tool (BET) was then used to generate a binary brain mask from the b = 0 image. Next DTIfit was used to independently fit the diffusion tensor to each voxel which yielded voxel-wise maps of FA, λ//and λ⊥.

### Tract-based Spatial Statistics (TBSS)

Voxelwise statistical analysis of FA was carried out using tract based spatial statistics (TBSS) within FSL [37] using the following routine. Firstly, the FA image of each subject was aligned to a 1 mm isotropic target FA image (FMRIB58_FA) using nonlinear registration by using a b-spline representation of the registration warp field [38]. The data was visually inspected to ensure accuracy of the transformations and then all of the aligned FA images were transformed into the 1 mm isotropic MNI152 template by means of by affine registrations [39]. A mean FA skeleton image representative of all tracts with a common centre was created from all subjects and individual subject FA images were projected onto this skeleton. Finally, voxelwise statistics across subjects (co-varying for age) were run for each point on the mean FA skeleton using permutation-based non-parametric testing (RANDOMISE - as implemented in FSL), using a 5000 permutation set [40] for contrasting differences between BD patients versus healthy controls. Family-wise error (FWE) correction [41] was used to correct the threshold for multiple comparisons across space and threshold-free cluster enhancement was employed to assess cluster significance [42]. The significant p-value with the FWE cluster corrected threshold was set at p<0.05. The transformation matrices that were created for registration of the FA maps were then applied to determine λ//and λ⊥ and white matter skeletons for each were created. Next, to further characterize observed changes in FA (ie whether changes were due to axon loss or demyelination) non-parametric testing of both λ//and λ⊥ was undertaken (separately) and mean values for FA, λ//and λ⊥ were derived from the significant clusters. Finally, the bipolar group was separated into bipolar I, bipolar II and bipolar spectrum sub-groups and the FA, λ//and λ⊥ indices for each sub-group were contrasted to the control group.

## Results

### Group Characteristics

The comparisons and characteristics between young people with BD and controls are reported in Table 2. Levene’s test indicated unequal variances for the HAMD, SOFAS, K-10 and BPRS, so Welch’s statistic was used for these variables. A one-way between subjects ANOVA revealed no significant between group differences for age [F (1, 95) = 1.29; p = .259] and IQ [F (1, 79) = 1.94; p = .168], however there was a significant difference in HAMD [F (1, 79) = 30.06; p<.001], SOFAS [F (1, 84) = 179.33; p<.001], K-10 [F (1, 82) = 39.98; p<.001], BPRS [F (1, 76) = 48.18; p<.001] and level of education [F (1, 94) = 22.55; p<.001], see Table 2.

**Table 2:** Patient demographic and instrument scores and their associated significance level.

	Controls (60.0% F: 40.0% M) mean [SD]	Bipolar Disorder (71% F: 29% M) mean [SD]	Significance (df) [p]
Age	24.05 [2.92]	23.03 [5.04]	F (1, 95) = 1.29 [.259]
HAMD	2 [2.20]	13.44 [10.70]	F (1, 79) = 30.06 [.001]
SOFAS	91.88 [3.39]	64.28 [11.51]	F (1, 84) = 179.33 [.001]
K-10	15 [5.64]	26.31[9.43]	F (1, 82) = 39.98 [.001]
BPRS	27.19 [4.03]	40.47 [9.47]	F (1, 76) = 48.18 [.001]
Education	15.21[1.92]	13.12 [2.22]	F (1, 94) = 22.55 [.001]
IQ	107 [7.46]	104.74 [6.69]	F (1, 79) = 1.94 [.168]

Notes: HAMD = Hamilton Depression Rating Scale; SOFAS = Social and Occupational Functioning Assessment Scale; K-10 = Kessler-10; BPRS = Brief Psychiatric Rating Scale; Education = Years of Education; IQ = Predicted Intelligence Quotient.

### DTI Tract Based Spatial Statistics

**Figure 1:**
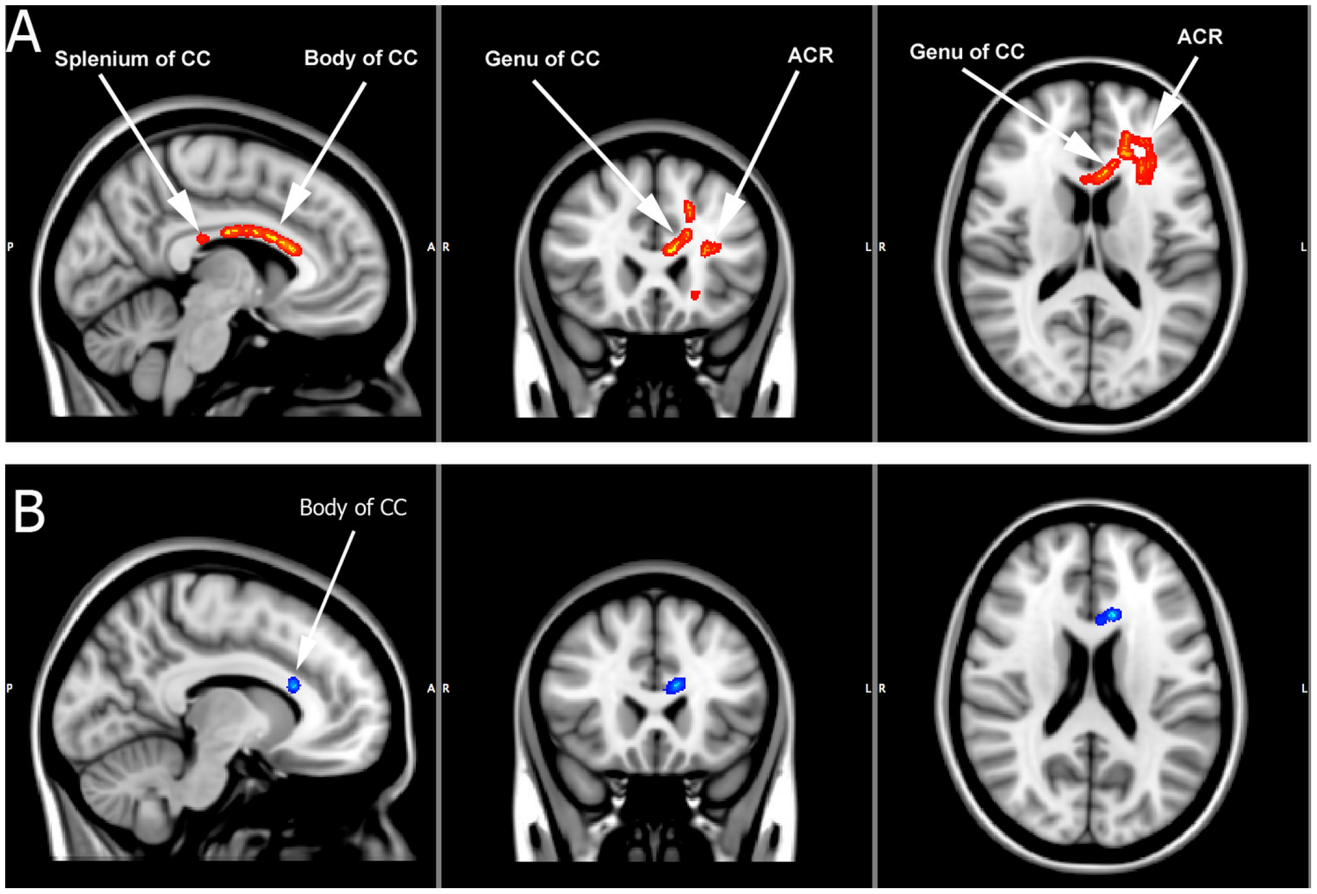
Entire BD group analysis. Panel A: Regions of significantly reduced FA in patients with BD (depicted in red/orange) compared to controls [CC = corpus callosum; ACR = anterior corona radiata]. All significant regions are cluster thresholded at p<.05 and corrected for multiple comparisons. Panel B: A region of significantly increased radial diffusivity within the body of the corpus callosum in patients with BD (depicted in blue) compared to controls.

**Figure 2:**
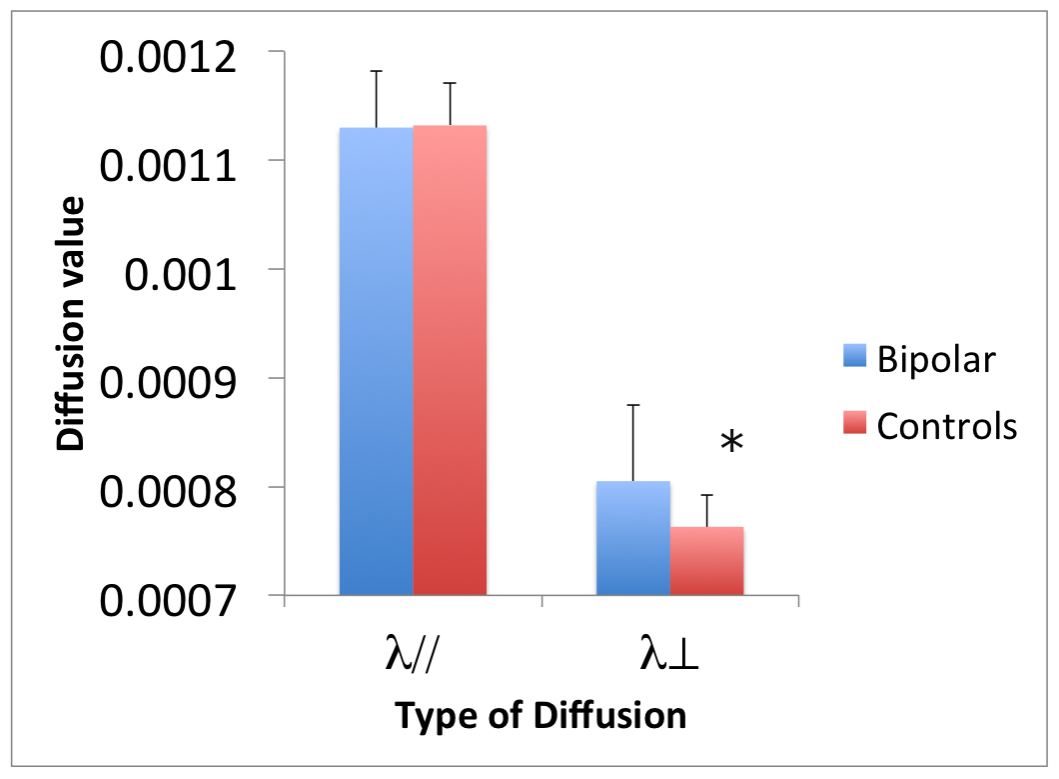
Measures of parallel and radial diffusivity for BD patients and controls. * p<.01.

Significantly decreased FA (corrected p<.05) was found in young people with BD within the corpus callosum. More specifically, significant white matter changes (corrected for multiple comparisons) were identified in the genu, splenium as well as the body of the corpus callosum. In addition significant white matter changes were also evident in the anterior and superior corona radiata (see Figure 1A and Table 3). Subsequent analysis revealed significantly increased λ⊥ (p<.01) in the body of the corpus callosum (with no changes in λ//) which suggests the changes in FA can be attributed to aberrations in radial diffusivity (Figure 1B and Figure 2).

**Table 3:** Clusters of significantly decreased FA in patients with bipolar disorder compared to healthy controls (anatomical loci are presented in MNI coordinates).

Region (All Bipolar patients)	Left/Right	x(mm)	y(mm)	z(mm)
Body of corpus callosum	right	15	−7	35
Body of corpus callosum	left	−15	−12	35
Splenium of corpus callosum	left	−18	−34	33
Genu of corpus callosum	left	−14	35	1
Anterior corona radiata	left	−22	30	−5
Superior corona radiata	right	25	−18	35
**Region (Bipolar I patients)**	**Left/Right**	**x(mm)**	**y(mm)**	**z(mm)**
Body of corpus callosum	right	8	8	15
Body of corpus callosum	left	−11	10	20
Genu of corpus callosum	left	−8	28	−2
Anterior corona radiata	left	−24	33	14

**Figure 3:**
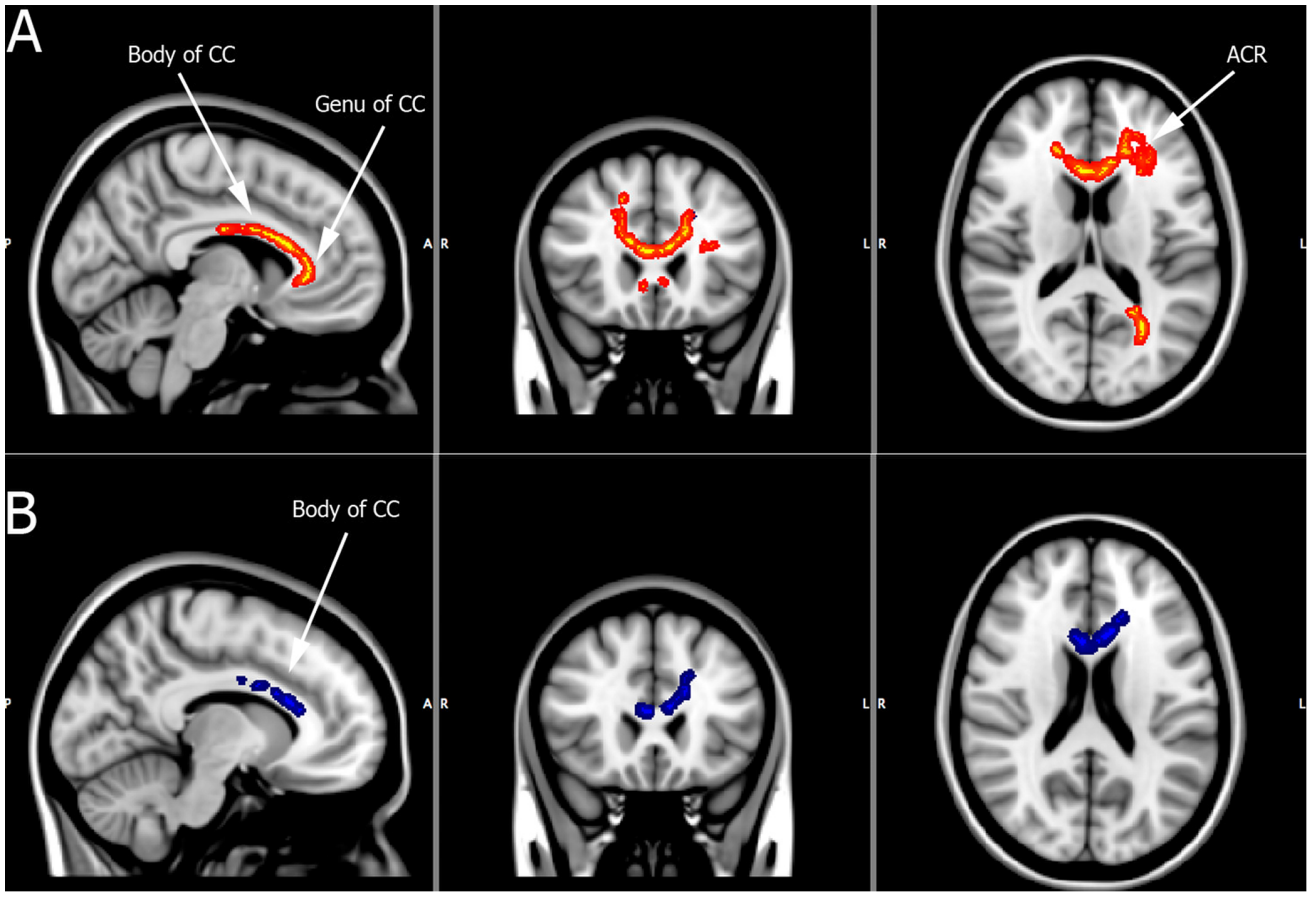
BD subgroup analysis. Panel A: Regions of significantly reduced FA for BDI patients (depicted in red/orange) compared to controls [CC = corpus callosum; ACR = anterior corona radiata]. All significant regions are cluster thresholded at p<.001 and corrected for multiple comparisons. Panel B: Significantly increased radial diffusivity within the body of the corpus callosum in BDI patients (depicted in blue) compared to controls.

In regards to the sub-group analysis only the bipolar I group was found to have significant differences when contrasted to controls after correcting the data for multiple comparisons. Significantly decreased FA (corrected p<.001) was observed in the genu and body of the corpus callosum along with the anterior corona radiata. In addition, significantly increased λ⊥ was also observed for the bipolar I group in the body of the corpus callosum (Figure 3).

## Discussion

This study reports significant changes in major white matter tracts from a large number of young patients in the early stages of BD. It adds to the growing literature that emphasises the extent to which these tract-based abnormalities are not confined to regions affected in older subjects with well-established illnesses. In the present study young patients with BD had significantly decreased FA within the genu, body and splenium of the CC. In addition significantly decreased FA was also present in the superior and anterior corona radiata. Further analysis was conducted on the aforementioned regions to elucidate the nature of the observed FA changes and a significant increase in λ⊥ was identified for the BD group within the body of the corpus callosum. When the BD group was separated into bipolar I, II and bipolar spectrum sub-groups, a similar pattern of significant changes in FA and λ⊥ was also present for the bipolar I group but not for the bipolar II and bipolar spectrum. Collectively, these results are consistent with the notion that BD is associated with discernable abnormalities in white matter integrity [5] and this is driven predominantly by the bipolar I phenotype.

In the current study, we investigated a younger cohort of people and thus our results are less likely to reflect changes that have developed in response to prolonged exposure to treatment or resultant from repeated affective episodes but rather be indicative of neurodevelopmental deviations or factors related to acute illness onset. A previous meta-analysis of white matter changes in BD (which excluded younger subjects) suggested that changes were largely localised to the right hemisphere and argued that this was consistent with studies of voxel-based morphometric and functional MRI studies [43]. The results in our current study for the most part corroborate previous DTI findings in older bipolar cohorts that have additionally reported aberrations in white matter integrity in the cingulum as well as the inferior and superior longitudinal fasciculi [1], [8]. In addition to white matter integrity, anatomical (non-DTI) changes within the CC in BD have also been reported [44], [45] and together with emerging DTI findings, this body of work supports the notion of disruptions to interhemisperic signal transduction in BD [46], a phenomenon that has been reported as far back as 1903 [47]. Notably, the aforementioned abnormalities bear similarities to recent findings reported in adolescents with BD as well as people “at risk” for BD [11]–[14].

The CC has rich interconnections with lateral aspects of the prefrontal cortex as well as medially with the rostral anterior cingulate cortex together with the insula [48]. The observed changes in the structural integrity along the length of the CC in conjunction with the superior and anterior corona radiata in our current study would most likely impact the transfer of information across a number of key anatomical regions including association projections from temporal and parietal cortex to the frontal lobes. Disruptions across these predominantly frontal networks are likely to culminate in the characteristic cognitive sequelae reported in BD, which include impairments to attention and executive functions.

To our knowledge this is the first study to investigate white matter changes across bipolar I, II and bipolar spectrum subgroups and report significant aberrations in λ⊥ in bipolar I patients. The increased λ⊥ reflects neuropathological changes within the body of the CC. This finding in the presence of a decreased FA (but normal λ//) indicates increased diffusion of water across the myelin sheath and thus is consistent with the interpretation of abnormalities in the myelin of white matter tracts [19]. Although DTI-based measures do not provide information regarding specific cellular changes they do reflect changes in the degree of myelination or axonal bundle coherence [49]. Our findings in conjunction with separate neuropathological studies that have reported reductions in the frontal oligodendrocyte populations [50] are intriguing and suggest that a glial dysfunction may underpin the core neuropathology of the disorder. Moreover, the evidence that oligodendrocytes are implicated in the disease process is further strengthened by a gene expression studies that have reported a down-regulation in myelin specific genes in this disorder [51], [52].

It is not yet clear that patients with BD have distinctly different white matter tract abnormalities from those with unipolar depression or schizophrenia. Indeed all three disorders share a subset of clinical symptoms [53], [54] as well as molecular and genetic aberrations [55], [56]. In reality, all of these studies are likely to be detecting common changes in specific tracts such as the anterior corona radiata that may be shared across these different diagnostic categories.

When clinical, neuroimaging and other biomarker studies are confined to young people in early stages of major psychiatric disorders; there is often far less clear separation of either clinical phenotypes or proposed biological illness markers. The detection of common neurodevelopmental changes (such as altered white matter tracts) is also consistent with the increasing recognition of at least some shared genetic risks across the major psychiatric disorders. It is also possible that detected abnormalities track other key behavioural or cognitive phenomena as distinct from specific diagnostic groups. For example, it is evident in the white matter literature relating to BD, the reported abnormalities may be linked to other key phenomena such as suicidal behaviour or aggression. The specific relationships with other key outcome phenomena – such as response to lithium therapy – require closer examination.

While the current study reports white matter abnormalities in a large cohort of young people in the early stages of illness, it has several limitations. The most obvious is the cross-sectional nature of the study. There is a need to track young people through various phases of illness to determine which changes represent likely prior vulnerability and differentiate these from illness-acquired or treatment-induced changes. A further limitation is that as subjects are early in their illness course, they inevitably vary in illness severity. We have included subjects from across the bipolar illness spectrum – that is, not limiting inclusion to those with discrete manic episodes or those with concurrent psychotic features. Larger studies of young people at these early stages are required to differentiate the possible mediating effects of the presence of other key features such as a discrete manic episode, prolonged psychotic symptoms or persisting cognitive impairment (although such sub-groups would still need to be matched for duration of illness and prior exposure to complex treatments). From a technical perspective it is important to indicate that our FA findings require careful interpretation around anatomical regions of crossing fibre bundles. In particular the anterior corona radiata is a region where several large white matter tracts (namely the uncinate fasciculus, inferior fronto-occipital fasciculus and the anterior thalamic radiation) overlap and in such instances the decrease in FA may not be associated with any white matter pathology. In our study however, we feel that this is unlikely especially given no significant FA changes were detected on the contralateral side. Finally, the potential confound of the effects of medication on the final FA results cannot be entirely discounted. However, it is important to highlight that the two main medications types that the patients were taking at the time of scanning were antipsychotics and lithium. In this regard, studies have reported that antipsychotic medications, have no significant effects on FA [57] and separate studies have shown that lithium has trophic effects on deep white matter and the oligodendrocyte network [58], [59].

In conclusion, our study builds on a small, emerging literature that reports the presence of white matter abnormalities within key brain regions in patients with BD. The results of our study highlight that young BD patients have discernable white matter pathology within the corpus callosum as well as frontal lobe white matter tracts. Moreover, these young patients also have significantly increased λ⊥ suggesting that a demyelinating process may underpin the pathophysiology and thus implicating oligodendrocytes and myelin as key factors. To our knowledge no other study has reported increased λ⊥ in a young BD cohort and thus our study emphasises the importance of investigating young patients at an early stage of their illness so as to better understand the underlying pathophysiology.
